# Zinc Burden Evokes Copper Deficiency in the Hypoalbuminemic Hemodialysis Patients

**DOI:** 10.3390/nu12020577

**Published:** 2020-02-23

**Authors:** Keizo Nishime, Morihiro Kondo, Kazuhiro Saito, Hisashi Miyawaki, Takahiko Nakagawa

**Affiliations:** 1 Nephrology Department, Rakuwakai Nijo Ekimae Clinic, 3Higashitoganoo-cho, Nishinokyo, Nakagyo Ward, Kyoto City 604-8414, Japan; 2 Nephrology Department, Rakuwakai Toji Minami Hospital, 1Nishikujo, Nanden-cho, Minami Ward, Kyoto 601-8441, Japan; morihirokondo@rakuwa.or.jp (M.K.); kazuhiro@rakuwa.or.jp (K.S.); 3 Graduate School of Home Economics, Kyoto Women’s University, 35Imakumano, Kitahiyoshi-cho, Higashiyama Ward, Kyoto 605-0926, Japan; Hisashimiyawaki@rakuwa.or.jp; 4 Department of Nephrology, Rakuwakai Otowa Hospital, 2 Otowa Chinji-cho, Yamashina, Kyoto 607-8062, Japan; takahikonakagawa@rakuwa.or.jp

**Keywords:** hemodialysis, zinc, copper, zinc-induced copper deficiency (ZICD), copper deficiency-induced myeloneuropathy (CDM)

## Abstract

Background: Recent research has focused on the roles of trace minerals such as zinc and copper. In 2017, oral zinc acetate was approved to treat zinc deficiency, and the next year, the Japanese Society for Clinical Nutrition developed the guidelines for diagnosis and treatment for zinc deficiency. Accordingly, hemodialysis patients began receiving zinc acetate when zinc deficiency was diagnosed. However, studies regarding the values of zinc and copper in hemodialysis patients are extremely poor, thus it remains unclear if the guidelines for healthy subjects can be applied to hemodialysis patients. Methods: We conducted a descriptive study, in which 132 patients were subjected to simply examine serum zinc concentration and its association with copper levels in hemodialysis patients (*N* = 65) versus healthy individuals attending a routine check-up (control group; *N* = 67) in our hospital. Analyses were performed with BellCurve for Excel (Social Survey Research Information Co., Ltd. Tokyo, Japan). Results: The distribution of zinc level in the hemodialysis group was distinct from that in the control group (*P* < 0.001). The zinc level was correlated with serum albumin concentration. Zinc concentration was also negatively correlated with serum copper level in both groups. In the hemodialysis group, the upper limit of zinc to avoid copper deficiency was 109.7 μg/dL, and the safety upper limit was 78.3 μg/dL. Conclusions: Hemodialysis patients exhibited a lower level of zinc concentration compared to normal healthy subjects. Since albumin binds to zinc as a carrier, low zinc levels could be attributed to lower level of serum albumin. Importantly, zinc and copper levels were inversely correlated, thus administration of oral zinc acetate could increase a risk for copper deficiency. It might be better to check both zinc and copper values monthly after prescribing zinc acetate.

## 1. Introduction

Zinc deficiency often develops clinical symptoms, including dermatitis or dysgeusia. In Japan, oral zinc acetate, a new drug, was approved against zinc deficiency in 2017. In the following year, the Japanese Society of Clinical Nutrition created a guideline for the diagnosis and the treatment of zinc deficiency. The guideline states that chronic disorders, including chronic kidney disease, liver diseases, and diabetes, often develop zinc deficiency (serum zinc concentration < 60–80 μg/dL), and zinc supplementation could improve any symptoms in those disorders. However, we should be careful to use oral zinc acetate, as excessive zinc supplementation could induce copper deficiency and develop several severe disorders, including pseudomyelodysplastic syndrome or subacute combined degeneration of spinal cord. In fact, the first report that zinc prescription causes copper deficiency was made by Prasad in 1978 [1]. 

Hemodialysis is a standard therapy to rescue patients with end stage renal disease. Blood filtration is the main process to remove uremic toxin under hemodialysis. However, as far as we know, there is no study on serum concentration of zinc in hemodialysis patients. Since the zinc drug was approved to be prescribed in 2017, it is urgent to know values of zinc and copper in hemodialysis patients. Here, we conducted a descriptive study examining zinc and copper levels in 65 patients receiving hemodialysis and 67 subjects attending a routine health check-up (control group). 

## 2. Patients and Methods

In total, 132 patients participated in this study, with 67 subjects attending a routine health check-up at Rakuwakai Toji Minami Hospital (control group) and 65 hemodialysis patients at Rakuwakai Nijo Ekimae Clinic (hemodialysis group). 

We compared zinc distribution and copper distribution between the hemodialysis group versus the control group. We also elucidated factors contributing zinc value using binomial logistic regression analysis. The regression line between zinc and albumin and the regression line between zinc and copper were calculated. We defined the upper/lower limit of zinc and copper based on the average ± 2SD of serum zinc or copper concentration. In turn, the upper/lower safety limit was set when both zinc and copper were within the upper and the lower limit. We clarified the zinc increasing period after prescribing zinc acetate (50 mg/bis, p.o.). Zinc and copper were estimated by the calorimetric method (SRL, Inc., Tokyo, Japan). Statistical analysis was performed with BellCurve for Excel (Social Survey Research Information Co., Ltd. Tokyo, Japan).

This study was approved by the institutional review board at Rakuwakai Marutamachi Hospital (Approval number: 2019-4). All procedures performed in studies involving human participants were in accordance with the ethical standards of the institutional review board at Rakuwakai Marutamachi Hospital (Approval number: 2019-4).

## 3. Results

**Figure 1:**
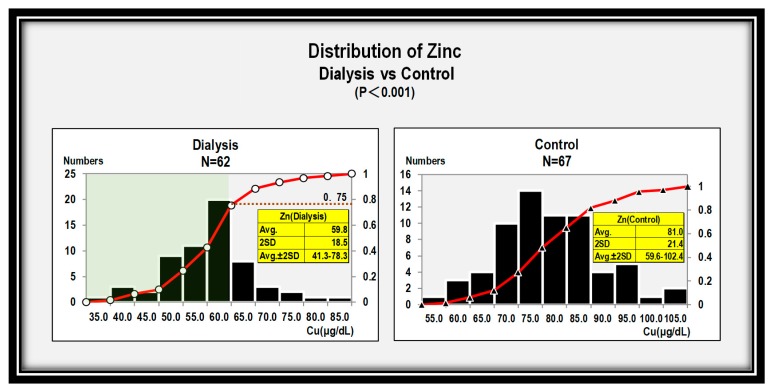
Significant differences in zinc level distributions are seen in the dialysis group versus the control group (*P* < 0.001).

First, we examined serum zinc concentration in patients of the hemodialysis group and the control group (Figure 1). It was found that there were significant differences in zinc level distributions between the hemodialysis group and the control group (*P* < 0.001), suggesting that the application of the control group value to the hemodialysis group would result in misdiagnosis. Zinc levels were below 60 μg/dL in 75% of hemodialysis patients according to cumulative frequency (red line). Zinc average ± 2SD was 41.3–78.3 μg/dL in the hemodialysis group, while it was 59.6–102.4 μg/dL in the control group (Figure 1).

**Figure 2:**
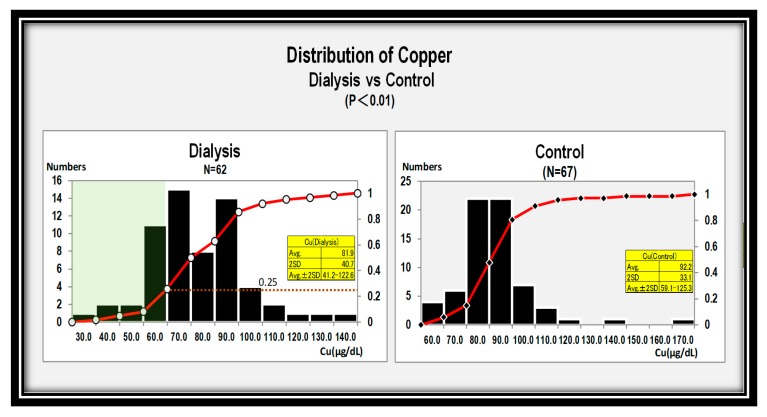
Distributions of copper levels in the dialysis group (left panel) and in the control group (right panel). Copper levels differed slightly between both groups (*P* < 0.01).

In turn, copper levels were slightly but significantly different between both groups (*P* < 0.01) (Figure 2). 

Since 75% of hemodialysis patients have lower zinc levels below 60 μg/dL, we divided the hemodialysis group into two subgroups based on zinc concentration ≥60 μg/dL or <60 μg/dL. According to binominal logistic regression analysis, albumin level was an explanatory variable (*P* < 0.05) for differentiation between the two subgroups. That means zinc value depends on albumin (Table 1).

**Figure 3:**
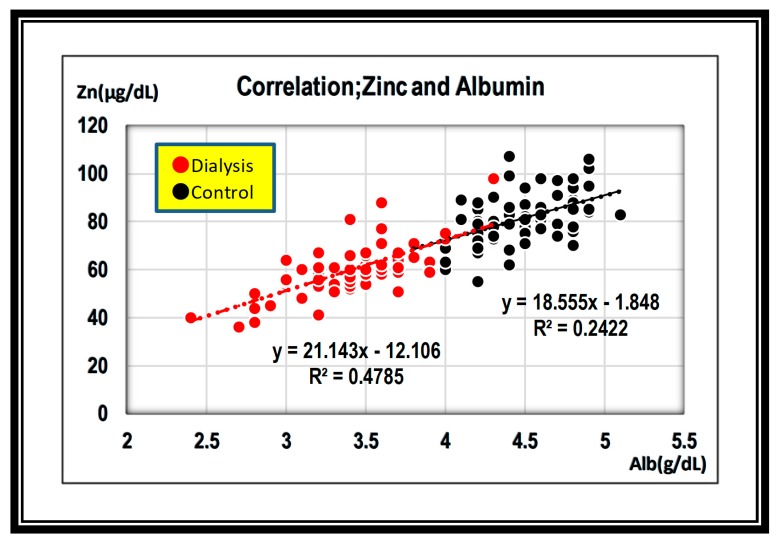
Regression line between zinc and albumin is positive in both groups. ●; dialysis group, ●; control group.

Next, we focused on albumin concentration, as albumin is thought to be a carrier for zinc [2]. We found that zinc concentration was positively correlated with albumin concentration in both groups. That indicated that zinc level was closely associated with albumin level. It was revealed that the zinc low value in the hemodialysis group results from low albumin value (Figure 3). 

**Figure 4:**
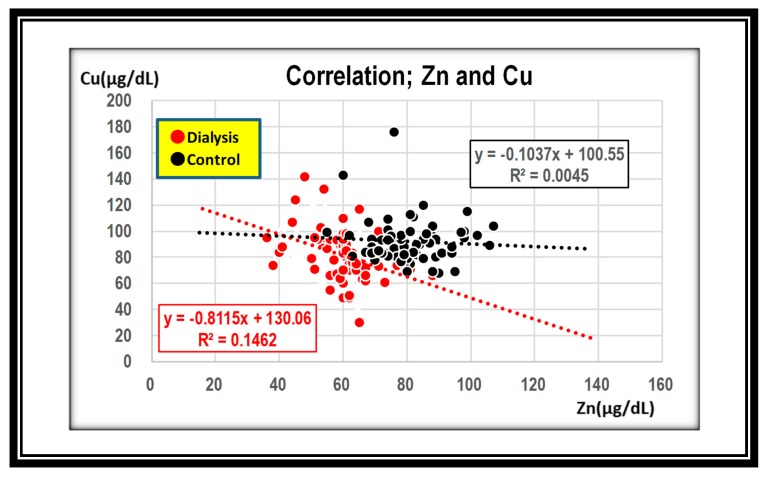
Zinc and copper values are negatively correlated in both groups. ●; dialysis group, ●; control group.

In terms of the relationship between zinc and copper levels, these were negatively associated in both groups. However, the slope was steeper in the hemodialysis group than in the control group, indicating that an increase in zinc levels reflects more rapid decline in copper levels in hemodialysis patients (Figure 4). 

**Figure 5:**
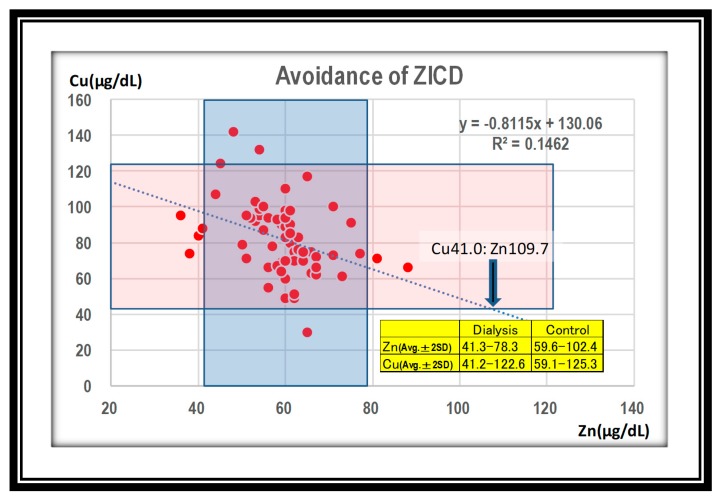
In the dialysis group, a zinc value over 109.7 μg/dL could cause copper deficiency. Squares show average. ± 2SD of zinc and copper in the hemodialysis group.

Next, we attempted to identify the critical point of copper deficiency by zinc loading. In the hemodialysis group, a zinc value above 109.7 μg/dL corresponded to a copper level below 41.0 μg/dL, which would be the copper deficiency in the hemodialysis group (Figure 5).

**Figure 6:**
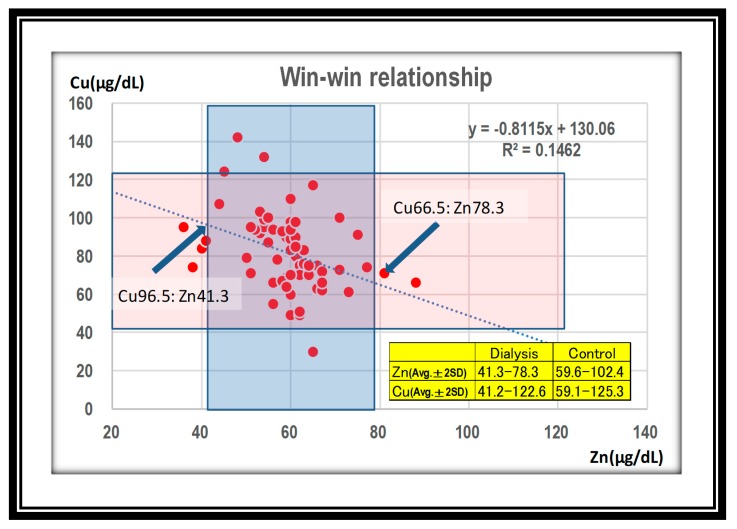
Two points are indicated for a win–win relationship of both zinc and copper in the dialysis group. Safety range of zinc is 41.3–78.3 μg/dL, and that of copper is 66.5–96.5 μg/dL.

Finally, we elucidated the ranges of a win–win relationship for zinc and copper. Two points were indicated for a win–win relationship of both zinc and copper in the hemodialysis group. The safety range of zinc was 41.3–78.3 μg/dL, and that of copper was 66.5–96.5 μg/dL (Figure 6).

Safety ranges mean causing no deficiency or over shoot in zinc and copper. The recommended safety ranges of zinc and copper in hemodialysis patients are shown in Table 2.

## 4. Discussion

Here, we found that serum zinc and copper concentrations were distinct between patients with hemodialysis and healthy subjects. Serum lower zinc concentration was associated with hypoalbuminemia, suggesting that we should be careful to evaluate zinc deficiency when albumin level is low. Importantly, zinc concentration was negatively correlated with copper concentration, suggesting overload of oral zinc acetate could cause copper deficiency, which could be manifested by neurological disorders, anemia, and others. 

A previous study documented that zinc levels were 60 to 120 μg/dL (average value ± 2SD) in 665 healthy Japanese (285 males and 380 females) [3]. Consistently, our data also showed zinc concentrations were 59.6–102.4 μg/dL (average value ± 2SD) in the control group. These data suggest that zinc concentration below 60 μg/dL would be considered as zinc deficiency in normal subjects. In turn, hemodialysis patients had lower zinc concentration at 41.3–78.3 μg/dL(average value ± 2SD), and the difference reached statistical significance (*P* < 0.001). In total, 75% of the hemodialysis patients showed zinc concentration below 60 μg/dL, thus these patients would be categorized as zinc deficient by the control average ± 2SD. These hemodialysis patients would be assumed to require zinc acetate supplementation (Figure 1). Unlike serum zinc levels, reported whole body zinc levels were not low among hemodialysis patients [4,5,6]. A next important finding is that there was a positive correlation between zinc and albumin levels in the hemodialysis group (Figure 3). This finding could be attributed to the fact that the current measurement system evaluates zinc ionized with a chelating agent bound to serum albumin. In addition, when inflammation results in low albumin, apparent hypozincemia occurs. Therefore, we should be careful to evaluate zinc concentration in the presence of hypoalbuminemia or active inflammation.

Copper concentration was also different between two groups. The average value for copper was 42.1 to 121.9 μg/dL in the hemodialysis group, whereas it was 59.1–125.3 μg/dL in the control group (Figure 2). This is our first documented study to show that zinc and copper levels were inversely correlated (Figure 4). Duncan et al. subjected 70 patients to examine the effect of zinc supplementation on copper deficiency using the stored records of prescriptions in Glasgow hospitals. One out of 70 patients developed zinc-induced copper deficiency (ZICD) with 135 mg zinc daily for nine months. However, a definitive conclusion for ZICD could not be made due to a lack of clinical information in the records, including the time of onset of symptoms, the reason for zinc prescription, and the response of withdrawal of zinc. In addition, they also found that plasma zinc concentration was low in the presence of hypoalbuminemia or systemic inflammatory response, suggesting that zinc deficiency was misdiagnosed by misinterpretation of low plasm zinc concentration due to hypoalbuminemia [7]. 

At the 64th Annual Meeting of the Japanese Society for Dialysis Therapy in 2019, copper deficiency was reported in up to 72% (13/18) of zinc-related presentations. Given these facts, it is important to know the safety zone in which both values are within appropriate ranges. Using the reverse regression line, we calculated the maximum zinc value that did not cause copper deficiency (defined as 41.0 μg/dL or less), and found that it was 109.7 μg/dL (Figure 5). Duncan et al. analyzed a database in Scotland (2000–2014, 8521 people). They found that high plasma zinc [>18 μmol/L (114 μg/dL)] and low plasma copper [<6 μmol/L (39 μg/dL)] were the predictive values to detect the patients named ZICD. They detected that 20 persons of 8521 satisfied two values. Investigating medical records and laboratory notes, fifteen patients of 20 were diagnosed with ZICD; 13 patients were found to suffer clinical symptoms, while hematological abnormalities were found in 13, and neurological abnormalities were in 11, respectively. Hematological abnormalities responded well to copper supplementation, but neurological abnormalities did not [8]. Their predictive values of zinc and copper were established on the basis of their experience and the literature. Although we derived our results from the regression line, the values are almost the same, which supports our claim. However, it would be safer if both zinc and copper concentrations were around average ± 2SD. In this regard, we would recommend a zinc level of 78.3 μg/dL and a level of 66.5 μg/dL in hemodialysis patients (Figure 6). In other words, a zinc value under 78.3 μg/dL is guaranteed to prevent ZICD. We chose this more conservative value because patients with pathological conditions such as gastrectomy [9,10,11,12,13], bariatric surgery [14,15], acrodermatitis enteropathica [16], Menkes disease, celiac disease [17], and nephrotic syndrome [18] are prone to copper deficiency. Caution related to hidden excess uptake of zinc from zinc fumes [19,20], zinc preparations [21,22], denture fixatives [23,24], and other unknown causes [25,26] is also necessary.

**Figure 7:**
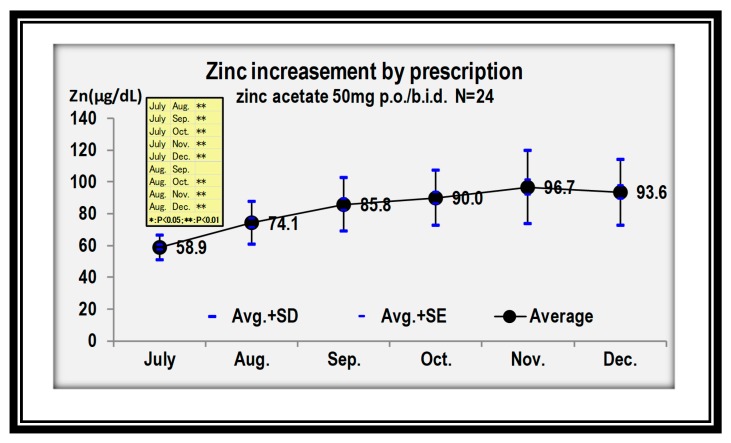
Increment of zinc by prescribing zinc acetate 50 mg/b.i.d, P.O. * *P* < 0.05. ** *P* < 0.01.

Zinc load-related copper-deficiency myelopathy has been reported in a hemodialysis patient [21]. CDM is now recognized as a new clinical entity in neurotoxicology [27,28]. We are currently treating a patient with myelopathy resulting from ZICD and will report our experience in the future. In the patient, we found that zinc levels increase significantly in the first month after initiating oral zinc acetate treatment (50 mg/b.i.d., p.o.) (Figure 7), thus monthly monitoring of zinc and copper levels would be required. 

Considering the risk of developing pseudomyelodysplastic syndrome or myeloneuropathy due to ZICD, an appropriate starting dose of zinc acetate might be 25 mg by mouth once daily. Zinc supplementation should be avoided in patients with possible copper malabsorption, such as those with a gastrectomy history or a zinc malabsorption condition such as celiac disease.

Finally, it is essential to understand the pathological conditions that are likely to cause pseudomyelodysplastic syndrome or myeloneuropathy resulting from ZICD. Mechanisms of ZICD are now well understood. Zinc stimulates enterocytes to produce chelator metallothionein, which shows stronger affinity to copper than to zinc. Copper binding to metallothionein then sloughs into the stool, resulting in copper deficiency [29]. Since metallothionein levels fall following zinc withdrawal, treatment with copper supplementation should continue for about 6 months until metallothionein-bound copper will not be lost in the stool. A supplement (copper sebacate; 3 mg/tablet) is available to treat ZICD.

## 5. Conclusions

Hemodialysis patients showed an apparent zinc deficiency. However, total body zinc, including tissues outside plasma, was sufficient. Low zinc levels resulted from the methodology, which mainly measured albumin-bound zinc. Because zinc and copper levels were inversely correlated, the safety upper limit for zinc to avoid copper deficiency was 78.3 μg/dL in hemodialysis patients. It is necessary to check both zinc and copper values monthly after prescribing zinc acetate. Finally, we described zinc-induced copper deficiency and copper deficiency -induced myeloneuropathy.

## Figures and Tables

**Table 1:** Explainable variable for two subgroups, binominal logistic reggresion analysis (*N* = 65).

	Zinc < 60 (μg/dL) *N* = 27		Zinc ≧ 60 (μg/dL) *N* = 38		
	Average	SD	Average	SD	
Age(Y)	73.9	11.9	71.0	11.6	
Heritage(Y)	8.5	9.3	6.0	4.2	
T-Cho(mg/dL)	147.9	26.2	151.2	28.7	
TP(g/dL)	6.2	0.6	6.4	0.5	
Alb(g/dL)	3.2	0.3	3.5	0.3	※※
CRP(mg/L)	0.5	0.9	0.3	0.7	
ALP(IU)	240.7	73.4	239.6	105.9	
WBC(×103)	5.3	1.7	5.9	2.1	
Hb(g/dL)	10.4	1.0	11.1	1.1	
MCV(fl)	93.7	6.9	94.1	5.3	
TSAT(%)	19.8	8.5	21.2	8.3	
Ferritin(ng/mL)	71.0	55.1	77.5	67.2	
Cu(μ/dL)	85.8	21.6	80.1	20.1	

※※ *P* < 0.01.

**Table 2:** Recommended Safety Ranges in hemodialysis patients.

Zn (μg/dL)	41.3~78.3
Cu (μg/dL)	66.5~96.5
